# Dissipation Pattern, Processing Factors, and Safety Evaluation for Dimethoate and Its Metabolite (Omethoate) in Tea (*Camellia Sinensis*)

**DOI:** 10.1371/journal.pone.0138309

**Published:** 2015-09-25

**Authors:** Rong Pan, Hong-Ping Chen, Ming-Lu Zhang, Qing-Hua Wang, Ying Jiang, Xin Liu

**Affiliations:** 1 Tea Research Institute, Chinese Academy of Agricultural Sciences, Hangzhou, China; 2 Graduate School of Chinese Academy of Agricultural Sciences, Beijing, China; 3 Key Laboratory of Tea Quality and safety & Risk Assessment, Ministry of Agriculture, Hangzhou, China; University of Vigo, SPAIN

## Abstract

Residue levels of dimethoate and its oxon metabolite (omethoate) during tea planting, manufacturing, and brewing were investigated using a modified QuEChERS sample preparation and gas chromatography. Dissipation of dimethoate and its metabolite in tea plantation followed the first-order kinetic with a half-life of 1.08–1.27 d. Tea manufacturing has positive effects on dimethoate dissipation. Processing factors of dimethoate are in the range of 2.11–2.41 and 1.41–1.70 during green tea and black tea manufacturing, respectively. Omethoate underwent generation as well as dissipation during tea manufacturing. Sum of dimethoate and omethoate led to a large portion of 80.5–84.9% transferring into tea infusion. Results of safety evaluation indicated that omethoate could bring higher human health risk than dimethoate due to its higher hazard quotient by drinking tea. These results would provide information for the establishment of maximum residue limit and instruction for the application of dimethoate formulation on tea crop.

## Introduction

Tea is considered as one of the most popular non-alcoholic beverages throughout the world. Nowadays, an increasing attention has been paid on tea because of its antioxidant, anticarcinogenic, and antimicrobial properties [1–3]. To prevent tea trees from being attacked by a variety of insects and diseases, organophosphorous pesticides were traditional pesticides widely used in the period of cultivation. However, due to its high mammalian toxicity, most of the organophosphorous pesticides have been forbidden or replaced by other pesticides. Only a few organophosphorous pesticides are still used in tea plantation, such as dimethoate [*O*, *O*-dimethyl-*S*-methylcarbamoylmethylphosphorodithioate]. Dimethoate is commonly used for control insects and mites by contact and stomach action [4]. Dimethoate has toxic effects on non-target organisms after application [5] and would be metabolized to omethoate in animals and plants [6]. Omethaote was found to be approximately 4 times more acute oral toxic to mammals than dimethoate[7].

Exposure of pesticides from plant-based foods might pose a potential threat to human beings and has become a dramatically global issue [8–9]. Lots of studies have put emphasis on pesticides behavior from environment and food chain using different extraction and quantification methods [10–11]. An increased awareness has been paid on pesticide residues in tea and various countries have revised their maximum residue limits (MRLs) of pesticides periodically. However, the MRL levels are highly variable. MRLs of dimethoate (usually considered as sum of dimethoate and omethoate) in tea are formulated at 0.05 and 1.0 mg/kg in the European Union and Japan, respectively. In the United States, dimethoate is regarded as the pesticide must not be detected, while there is no MRL of dimethoate in China. Such lack of global harmonization in pesticide MRLs constitutes a barrier to trade and causes severe economic losses of tea exporters [12].

Tea cultivation and manufacturing usually result in a significant decrease in pesticides. Meanwhile the leaching efficiency of pesticides during brewing is one of the key factors of human exposure for pesticides by drinking tea. Thus, numbers of literatures concerning residue levels of many pesticides in fresh tea leaves, processed tea, and their infusion have been reported [13–15]. Due to the effects of evaporation, photolysis, and growth dilution [16], field dissipation plays the most important role in chemical degradation. Tea manufacturing also does contributions to residue reduction [17–18]. Pesticides remained in made tea would be transferred into tea infusion and then exposed for humans. The leaching efficiency differs from pesticide to pesticide [19]. Such studies provide a more accurate understanding of pesticide and are helpful in risk assessment for tea drinking.

The dissipation behavior of dimethoate during tea cultivation was found to be fitted with first-order kinetics [20–21]. However, there is no literature concerning about the fate of dimethoate and its metabolite under each step of tea manufacturing, except for Sood, et al. [22] who reported the effects of green and black tea manufacturing on dimethoate. With high water solubility, dimethoate and omethate in made tea are easy to transfer into tea infusion and adsorbed by tea drinkers. Transfer rate of dimethoate has been reported ranging from 6.4% to 91% in tea (*Camellia Sinensis* L.) [23], mate tea [24], peppermint tea [25], and stinging nettle tea [26]. In this study, the dissipation behavior of dimethoate and its oxon metabolite (omethoate) during tea planting, manufacturing, and infusion was investigated and safety evaluation by tea drinking was also carried out. These results would provide information for establishment of MRL and instruction for the application of dimethoate formulation on tea crop.

## Materials and Methods

### Materials

Commercial dimethoate (40% EC) was purchased from Tenglong Biological Pharmaceutical Co., Ltd. (Jiangsu, China). The standard of dimethoate and omethoate were purchased from Agro-Environment Protection Institute (1000mg/L, Tianjin, China). Acetonitrile, dichloromethane were both analytical-grade and were obtained from Merk (Darmstadt, Germany). Analytical-grade sodium chloride (NaCl), anhydrous sodium sulfate (Na_2_SO_4_), and anhydrous magnesium sulfate (MgSO_4_) (Zhejiang Medicine, Zhejiang, China) were baked for 3 h at 650°C prior to use. Primary-secondary amine (PSA), octadecylsilane (C_18_), and graphitized carbon black (GCB) (Agela, Tianjin, China) were used as sorbents for purification. Ultrapure water was made in the laboratory by Milli-Q water system (Bedford, MA, USA).

### Field trials

This trial was conducted in experimental tea plantation (variety, Longjing 43) located in Tea Research Institute, Chinese Academy of Agricultural Sciences, (TRI CAAS) Hangzhou, China, from July to August, 2014. This study is permitted by TRI CAAS. No protected land area or wildlife was used and no endangered or protected species was involved. Before the trial, fresh tea leaves collected in this plantation were determined free of dimethoate and omethoate. The plantation was divided into 6 plots with 100 m^2^ each, leaving one row of bushes as separation guards. 5 plots were applied with commercial dimethoate dilutions (1:400) by a hand-operated knapsack sprayer for replication, whereas the remained plot, which was sprayed with similar amount of water, was considered as control.

### Sampling and sample preparation

**Fig 1:**
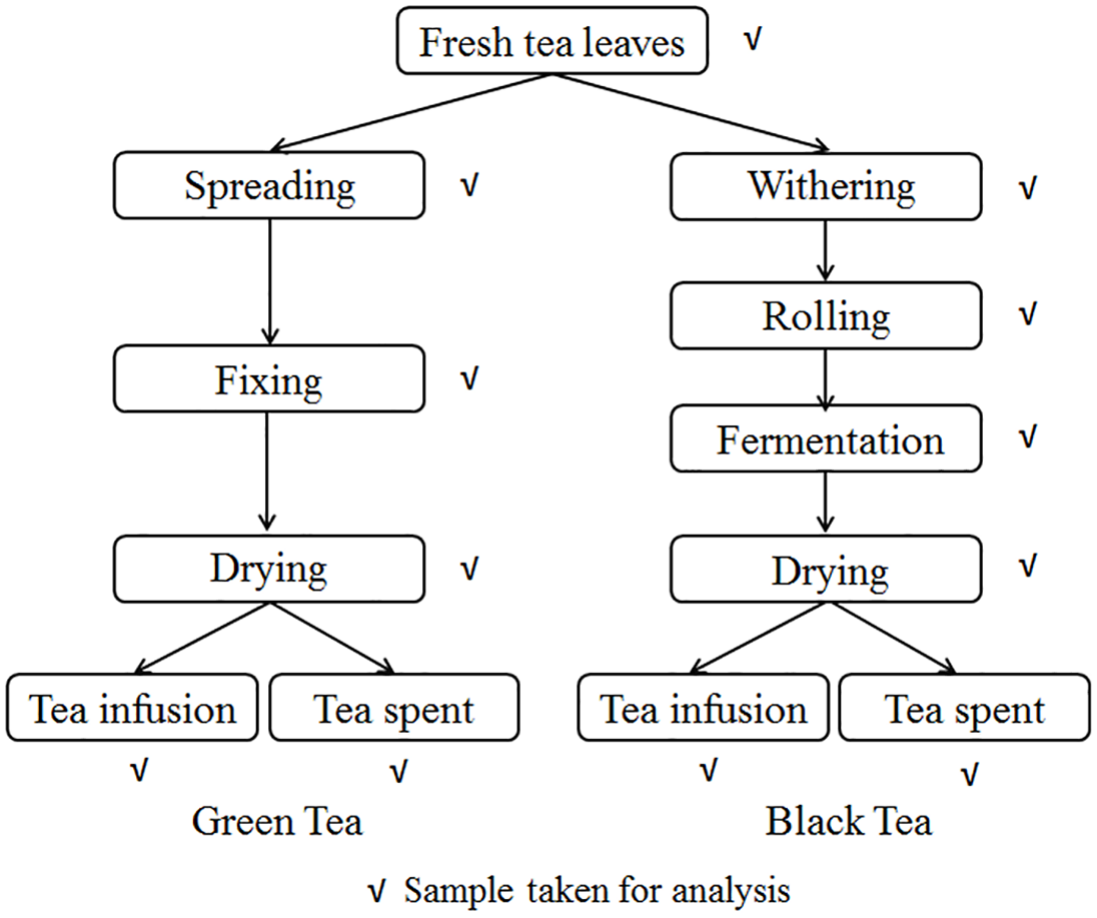
The flow chart of traditional manufacturing process of green tea and black tea.

The fresh tea leaves consisting of one bud and two leaves were picked from both treated and control plots at 0 (2 h), 1, 2, 3, 4, 5, 6, 7, 10, 14, 21 d after application of dimethoate formulation. Fresh leaves picked on days 1 and 3 were submitted to traditional green tea (spreading, fixing, drying) and black tea (withering, rolling, fermentation, drying) manufacturing steps immediately (Fig 1), which is similar to the procedure described by our previous study [27]. During each stage of tea manufacturing, samples were collected and analyzed. All the samples mentioned above were detected on the same day of collection prior to storage. Both green tea and black tea was submitted to further brewing. 3 g of made tea was brewed with 150 mL boiled water. After infusing 5 min, the infusion was introduced into baker, while the spent leaves were left. Both tea soup and spent were cooled and then analyzed.

### Analytical method

#### Extraction

2 g of analyzed samples (except fresh tea leaves with water content > 70%) was soaked with 1 mL ultrapure water in advance, and then extracted with 10 mL of acetonitrile by homogenization for 2 min at 12000 rpm. Subsequently, 5 g of NaCl and 1 g of MgSO_4_ were added. Sample was vortexed for 30 s followed by a centrifugation for 10 min at 5000 rpm. For the fresh leaves collected on and after day 10, 4 g of tea samples was used and the extraction solvent was doubled. After centrifugation, 10 mL of the supernatant was transferred and evaporated to 2 mL. Spent tea leaves after brewing were all cut into pieces and transferred into centrifuge tube, then extracted with 15 mL of acetonitrile by homogenization. Residues in tea soup (50 mL, dissolved with 10% NaCl) were extracted by 50 mL of dichloromethane. The organic phase was filtered through 15–20 g of Na_2_SO_4_ and evaporated into 2 mL.

#### Clean-up

2 mL of the supernatant was transferred into a 5 mL centrifuge tube, which contains 200 mg of PSA, 200 mg of C_18_, 50 mg of GCB, and 100 mg of MgSO_4_, then mixed well by votexing. After centrifugation (4000 rpm, 10 min), the supernatant was filtered through 0.22 μm membrane into sample vials. Extractions of tea infusion with a few impurities would transfer through membrane into sample vials without clean-up.

#### GC analysis

Dimethoate and omethoate were determined by gas chromatography coupled with flame photometric detector (Agilent, America)and HP-1701 column (30 m × 0.53 mm × 1μm, Agilent, Shanghai, China). The temperature of injection and detector were 230°C and 245°C, respectively. Oven temperature was maintained at 80°C for 1 min, then raised to 230°C at 25°C/min for 23 min. 99.99% purity nitrogen was employed as carrier gas, the flow rate was 5.0 mL/min. The injection volume was 1 μL in spiltless mode. Quantification was conducted by external standard method using matrix-matched standard solution.

### Data analysis

Generally, the dissipation behavior of a pesticide was evaluated by the first-order kinetics [28–29]. In this study, Sigmaplot 12.5 was applied for dynamic fitting on the basis of Eq (1), where *k* presents the corresponding rate constant of dimethoate, and *C*_*t*_ and *C*_0_ present the concentration of dimethoate (mg/kg) at time *t* and 0, respectively. Half-life (*T*_1/2_) of dimethoate (d) was calculated by Eq (2).

$$C_{t}=C_{0}\times e^{–kt}$$ (1)

$$T_{1/2}=ln\;2/k$$ (2)

The effects of tea manufacturing were performed by processing factor (PF) [30], which was calculated by Eq (3) as follows:

$$PF=C_{1}/C_{2}$$ (3)

where *C*_1_ and *C*_2_ stand for the concentration of a chemical in processed products and raw materials (mg/kg), respectively.

Transfer or remained rates of dimethoate and its metabolite during infusion were calculated by Eq (4) or Eq (5).

$$Transfer\;rate(\%)=m_{2}/m_{1}\times 100$$ (4)

$$Remained\;rate(\%)=m_{3}/m_{1}\times 100$$ (5)

where *m*_*1*_, *m*_*2*_, *m*_*3*_ present the concentration of residues in made tea, tea infusion, and spent tea leaves (mg/kg), respectively.

The estimated daily intake (EDI) of a pesticide was calculated as follows:

EDI=C×Transfer rate(%)×K (6)

Where *C* is the concentration of a pesticide in made tea (mg/kg), *K* is the average consumption rate of tea and was referred as 10 g^-1^ bw day^-1^.

Hazard quotient (HQ) was used for long-term risk assessment.

HQ (%) = EDI / ADI × 100 (7)

where ADI was the acceptable daily intake of a pesticide. It was 2 and 0.3 μg kg^-1^ bw day^-1^ for dimethoate and omethoate, respectively.

## Results and Discussion

### Method validation

Good linearities were obtained with correlation coefficients of more than 0.9979 and 0.9708 for dimethoate and omethoate, respectively. The limits of quantification (LOQs) in fresh tea leaves were found to be 0.008 mg/kg and 0.03 mg/kg for dimethoate and omethoate, respectively. LOQs of dimethoate were 0.0004 mg/L in tea infusion and 0.05 mg/kg in other matrices, whereas LOQs of omehtaote were0.0008 mg/L in tea soup and 0.05–0.10 mg/kg in other matrices. These values of LOQ have reached the analytical sensitivity in our study, although they are a little high.

The efficiency of this method was evaluated by spiking various tea matrices with dimethoate and omethoate in two different levels. Recoveries of dimethoate for fresh tea leaves, made tea, and spent leaves ranged from 80.4% to 85.2% with relative standard deviations (RSDs) <5%. All these values of recovery indicated good method accuracy and repeatability. Recoveries of omethoate for fresh tea leaves, made tea, and spent leaves ranged from 55.2% to 59.1%, with an acceptable RSDs<7%. According to European Council N° SANCO 12495–2011 [31], the recovery of omethoate (<70%) could also be acceptable for its consistency. In order to reflect the actual level of omethoate, mean recovery (57%) was used to correct the data in this study, since there is no significant difference of recoveries among various tea matrices (P = 0.1610). However, for tea soup, the recovery was quite low (<30%) so that omethoate in tea soup was not under consideration in transfer rate calculation.

### Dissipation behavior on tea shoots

**Fig 2:**
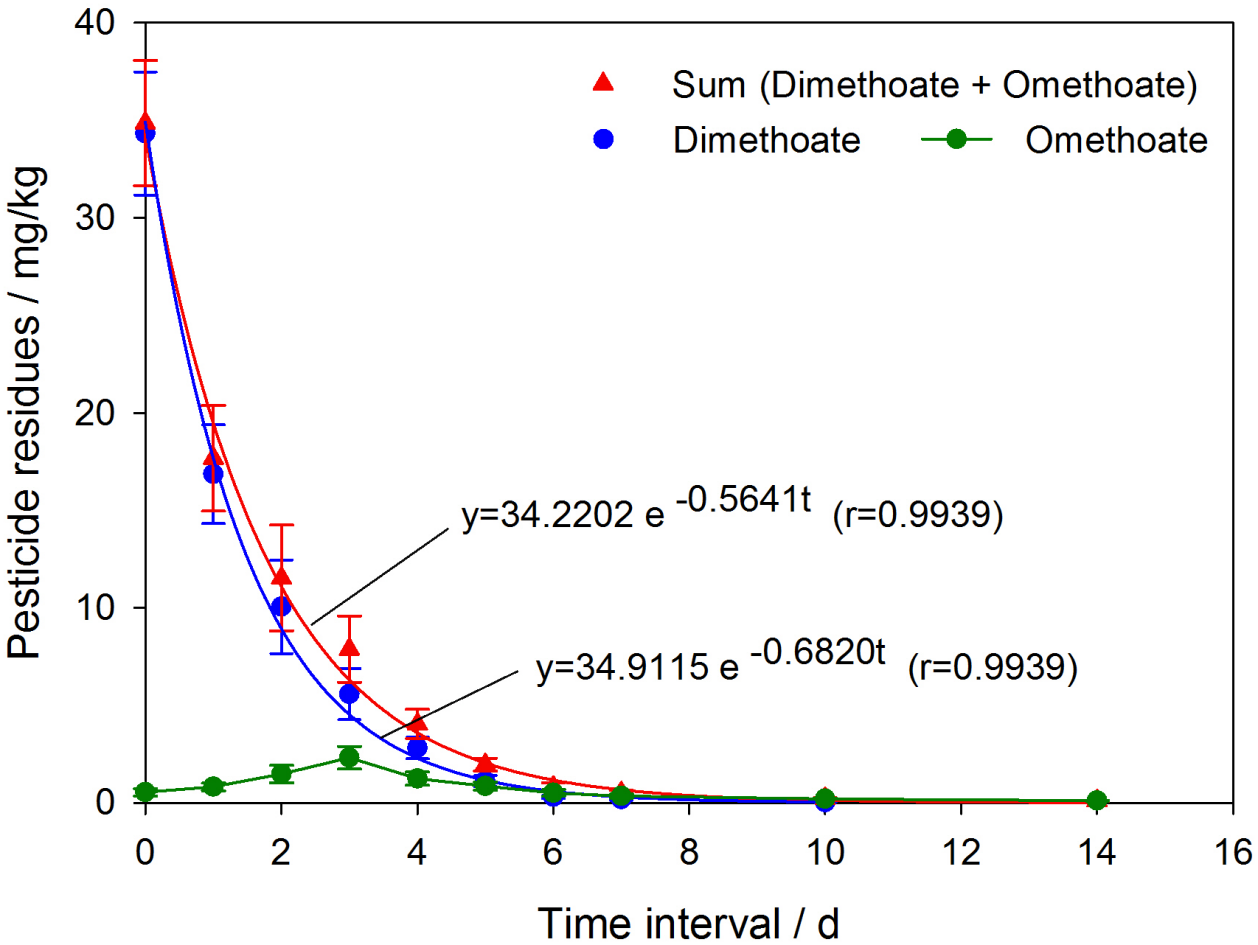
Dissipation of dimethoate and its metabolite on fresh tea leaves. (The concentrations of omethoate have been corrected by mean recovery of 57%).

The residue levels of dimethoate and its metabolite are presented in Fig 2. Dissipation behavior of dimethoate followed the first-order kinetic (*C*_*t*_ = 34.9115 × e ^−0.6820t^) with a high correlation coefficient of 0.9939. The initial deposits of dimethoate were 34.33 mg/kg and then dissipated rapidly to 5.57 mg/kg on day 3 after application, resulting in a great reduction of 83.8%. Subsequently, it showed a quite slow dissipation and could not be detected on day 14. The half-life of dimethoate on tea shoots was 1.08 d, which was similar to the result (0.95 d) by Chen & Wan [20]. Persistence of dimethoate has also been studied earlier by Utture, et al. [32] on pomegranate and it was found that dimethoate in pomegranate whole fruits with a half-life of 5.75–7 d, which was much longer than our present study. On onion, the half-life of dimethoate was investigated to be 2 d [33], which was also a little higher than the present study. This may be due to the differences in matrix, agricultural conditions and climate. Wherein, growth dilution maybe the dominating factor leading to the rapid dissipation of dimethoate in tea shoots [34–35].

Before application, the formulation of dimethoate had been analyzed and results demonstrated that there is only few omethoate (0.1%) in it. However, the concentration of omethoate increased to 2.30 mg/kg on day 3. This indicated that dimethoate, applied in tea plantation, was able to be degraded into omethoate, resulting in a considerable amount of omethoate remaining in tea shoots. The residues gradually decreased thereafter and could not be detected on day 21 after application.

As a whole, total residues of parent compound dimethoate and its metabolite omethoate present a similar reduction with dimethaote from the initial deposits of 34.86 mg/kg (Fig 2). The dissipation behavior could be described by the following first-order kinetic equation: *C*_*t*_ = 34.2202 × e ^−0.5641t^(r = 0.9939). The half-life of total residues on tea shoots was 1.27 d, which is 18% longer than that of parent compound dimethoate. This indicated that omethaote, as metabolite of dimethoate, should be taken into account in dimethoate dissipation and risk assessment.

### Residues of dimethoate and omethoate during tea manufacturing

Concentrations and processing factors of dimethoate and its metabolite during tea manufacturing were presented in Table 1. Residues of dimethoate increased to 35.51 (13.44) mg/kg and 23.84 (9.48) mg/kg after green tea and black tea manufacturing respectively, from the same initial deposits of 16.85(5.57) mg/kg on day 1 (day 3). PF values of dimethoate changed from 1.06–1.08 to 2.11–2.41 and from 1.29–1.34 to 1.41–1.70 during each steps of green tea and black tea manufacturing, respectively. These results indicated that dimethoate was preferred to dissipate during black tea manufacturing, which is consistent with Hou, et al. [12].

**Table 1:** Concentrations and processing factors of dimethoate and its metabolite during green tea and black tea manufacturing.

Style	Stages	Percentages of dry matter (%)(PF)	Dimethoate concentration(PF)		Omethoate concentration ^a^(PF)		Sum concentration ^a^(PF)	
			Day 1 ^b^	Day 3 ^b^	Day 1 ^b^	Day 3 ^b^	Day 1 ^b^	Day 3 ^b^
Green tea	Fresh leaves	21.30±0.28(–)	16.85±2.54(–)	5.57±1.31(–)	0.82±0.20(–)	2.30±0.58(–)	17.67±2.72(–)	7.87±1.70(–)
	Spreading	25.17±0.52(1.18)	18.24±2.78(1.08)	5.91±0.23(1.06)	1.68±0.33(2.09)	1.08±0.17(0.47)	19.92±2.87(1.13)	6.98±0.25(0.89)
	Fixing	40.28±1.37(1.89)	27.39±3.71(1.63)	8.50±1.29(1.53)	1.88±0.45(2.33)	1.82±0.51(0.79)	29.99±4.54(1.70)	10.33±1.60(1.31)
	Drying	96.96±0.19(4.55)	35.51±1.99(2.11)	13.44±1.22(2.41)	0.93±0.16(1.15)	1.46±0.16(0.63)	36.44±2.09(2.06)	16.03±1.78(2.04)
Black tea	Fresh leaves	21.30±0.28(–)	16.85±2.54(–)	5.57±1.31(–)	0.82±0.20(–)	2.30±0.58(–)	17.67±2.72(–)	7.87±1.70(–)
	Withering	37.05±0.73(1.74)	21.76±1.92(1.29)	7.49±0.62(1.34)	2.52±0.45(3.13)	2.35±0.59(1.02)	24.28±2.09(1.37)	9.84±1.08(1.25)
	Rolling	42.41±0.14(1.99)	24.04±1.00(1.43)	8.21±0.85(1.47)	2.75±0.28(3.41)	2.80±0.51(1.21)	26.79±1.18(1.52)	10.99±1.14(1.40)
	Fermentation	42.55±1.62(2.00)	22.98±1.74(1.36)	7.66±0.69(1.38)	2.29±0.43(2.83)	2.62±0.39(1.14)	26.19±2.20(1.48)	9.97±0.47(1.27)
	Drying	97.05±0.18(4.56)	23.84±4.30(1.41)	9.48±0.48(1.70)	1.02±0.23(1.26)	1.74±0.30 (0.75)	24.86±4.51(1.41)	9.96±0.55(1.27)

^a^The concentrations of omethoate have been corrected by the mean recovery (57%).

^b^ Day 1 and day 3 present the date on which tea manufacturing were conducted.

PF<1 indicates a reduction whereas PF>1 indicates a concentration of the residue after processing [36]. PF values in this study, higher than 1, suggested that tea manufacturing leading to a concentration of dimethoate. However, the residues of dimethoate decreased actually, because there is a weight concentration (up to 4.56 times) during the manufacturing due to water evaporation. Comparing the difference of PFs for dimethoate and dry matter concentration, we found that drying did the greatest contribution to the dimethoate dissipation. This finding is in agreement with Gupta & Shanker [13]who reported that the loss of acetamiprid (8–13%) and imidacloprid (9–13%) were quite significant during drying among all steps in black tea manufacturing. The loss of residue would mainly attribute to factors of degradation, evaporation, and co-distillation [22]. PF ratio of dimethoate and dry matter concentration after withering (0.74–0.77) was much lower than spreading (0.90–0.92), indicating larger loss of dimethoate was occurred during withering than spreading. Withering was also implied to be the key factor in large loss of dimethoate in black tea manufacturing than green tea manufacturing, which is in agreement with Hou, et al. [12]. Additionally, PF values of made tea on day 3 were much higher than day 1, when the fresh tea leaves underwent the same treatments. This phenomenon may be explained the distribution of dimethoate. Dimethoate has a good penetrating ability due to its hydrophilic property [37]. Residues, penetrated into the interior of the cuticle, took greater effort to achieve the same degree of dissipation.

PF values of omethoate in green tea and black tea were 0.63–1.15, 0.75–1.26, respectively. During tea manufacturing PF values showed an increasing to maximum before drying, and then decreased to 1.15–1.26 on day 1 whereas 0.63–0.75 on day 3. Compared with the change of dry matter percentages during each stage of tea manufacturing, PF values of omethoate before drying on day 1 was higher, which indicated that there was an increase of omethoate indeed. Dimethoate could be degraded into omethoate, and the metabolite (omethoate) underwent dissipation as well during tea manufacturing. Opposite to dimethoate, PF values of omethoate on day 1 was higher than day 3 during both green tea and black tea manufacturing. This may be related to the initial deposition of dimethoate and omethoate on fresh tea leaves. Higher deposition of dimethoate led to more degradation of omethoate, resulting in the change of omethoate with low deposition less significant.

Additionally, total residues of parent compound dimethoate and its metabolite omethoate increased to 16.03–36.44 mg/kg after green tea manufacturing, while it increased to 9.96–24.86 mg/kg after black tea manufacturing, from the same initial deposits (17.67, 7.87 mg/kg on days 1 and 3, respectively). PF values of the total residues increased from 0.89–1.13 to 2.04–2.06 during green tea manufacturing, whereas it showed an increase from 1.25–1.37 to 1.40–1.52 after rolling and decreased to a final value of 1.27–1.41 thereafter during black tea manufacturing. PF ratios between dry matter and corresponding total residues were all higher than 1, indicating that total residues of dimethoate and omethoate decrease with each stage of tea manufacturing indeed. The losses of total residues were sum up to 54.7–55.3% and 69.1–72.2% during green tea and black tea manufacturing, respectively. PF ratios between dry matter and corresponding total residues during drying ranged from 2.21 to 3.59, which is much higher than that of any other stages (1.04–1.57), in both green tea and black tea manufacturing. This indicated that drying was the most important factor in residues dissipation during tea manufacturing.

### Transfer rates from made tea to infusion

Residues of dimethoate and omethoate in made tea, tea infusion as well as spent leaves were presented in Table 2. The transfer rates of dimethoate to infusion were 80.5–81.9% and 81.0–82.5% from green tea and black tea respectively, whereas 12.7–16.2% of the residues remained in the spent leaves. Since the recovery of omethoate in tea infusion was too low (<30%), the transfer rate was beyond calculation. Omethoate remained in spent leaves ranged from 15.1% to 16.7%. It could be inferred that about 85% of omethoate was transferred from made tea into brewing.

**Table 2:** Residues of dimethoate and its metabolite in made tea, tea infusion, and spent tea leaves, and their transfer into tea infusion.

Pesticide	Day after spraying	Matrix	Residues^a^			Transfer (%)	Remained(%)
			Made tea(mg/kg)	Tea infusion^b^(mg/L)	Spent leaves(mg/kg)		
Dimethoate	1	Green tea	35.51±1.99	0.58±0.06	5.18±0.39	81.9	14.6
	3		13.44±1.22	0.22±0.01	1.71±0.15	80.5	12.7
	1	Black tea	23.84±4.30	0.39±0.04	3.87±0.34	82.5	16.2
	3		9.48±0.48	0.15±0.01	1.28±0.07	81.0	13.5
Omethoate ^c^	1	Green tea	0.93±0.16	–	0.14±0.02	–	15.1
	3		1.46±0.16	–	0.23±0.04	–	15.7
	1	Black tea	1.02±0.23	–	0.17±0.02	–	16.7
	3		1.74±0.30	–	0.27±0.04	–	15.5

^a^ Values presented by the mean ± SD (n = 5).

^b^ Recovery of omethoate was too low (<30%), thus the residues in tea infusion was not under consideration in the transfer rates calculation.

^c^The concentrations of omethoate have been corrected by the mean recovery (57%).

Comparing the remained percentages in spent leaves, no significant difference was observed between dimethoate and omethoate (P = 0.1441). This indicated that dimethoate and omethoate presented a similar ratio in transfer rate. The transfer rate of pesticide from made tea to infusion mainly depends on its water solubility (*W*_*s*_) and octanol-water partition coefficient (*K*_*ow*_) [38–40]. Wan, et al. [40] also found that the extraction rates of pesticides with *W*_*s*_ more than 170 mg/kg is no longer so sensitive to the change of *W*_*s*_. Thus, dimethoate and omethoate with quite high *W*_*s*_ [*W*_*s(dimethoate)*_ = 39800 mg/L; *W*_*s(omethoate)*_ = 10000 mg/L] and low *K*_*ow*_ [*K*_*ow (dimethoate)*_ = 0.704; *K*_*ow (omethoate)*_ = −0.74] is hydrophilic in nature, both showing high transfer rates during tea brewing. However, Ozbey & Uygun [25–26] observed higher dimethoate transfer rate of 91% in peppermint tea and 86–89% in thyme and stinging nettle tea, whileJaggi, et al. [23] reported that 26.2–32.5% of the dimethoate was transferred into tea (*Camellia Sinensis* L.) infusion and only 6.4% of dimethoate was found to be transferred during mate tea brewing process [24].

### Safety evaluation

Since human exposure of pesticide residues in tea is mainly underwent through drinking tea infusion, transfer rates of pesticides should also be taken into consideration for safety evaluation. Estimated daily intake (EDI) and hazard quotient (HQ) of dimethoate & omethoate were presented in Table 3. EDI and HQ of dimethoate and omethoate on days 5–14 were estimated based on the mean dissipation and mean transfer rates of residues on days 1 and 3.

**Table 3:** Estimated daily intake (EDI) of dimethoate and its metabolite and risk assessment in tea.

Time interval(d)	Pesticide	Green tea				Black tea			
		Residue^a^ (mg/kg)	Transfer^a^(%)	EDI(μg kg^-1^bw day^-1^)	HQ(%)	Residue^a^(mg/kg)	Transfer^a^(%)	EDI(μg kg^-1^bw day^-1^)	HQ(%)
1	Dimethoate	35.51	0.82	4.85	242.36	23.84	0.83	3.28	163.90
	Omethoate	0.93	0.85	0.13	43.87	1.02	0.83	0.14	47.20
					HQs:286.22				HQs: 211.10
3	Dimethoate	13.44	0.81	1.80	90.16	9.48	0.81	1.28	63.99
	Omethoate	1.46	0.84	0.21	68.38	1.74	0.85	0.25	81.68
					HQs:158.54				HQs: 145.67
5	Dimethoate	2.18	0.81	0.30	14.77	1.50	0.82	0.21	10.25
	Omethoate	1.69	0.85	0.24	79.26	1.16	0.84	0.16	54.20
					HQs:94.03				HQs: 64.45
7	Dimethoate	0.34	0.81	0.05	2.28	0.23	0.82	0.03	1.58
	Omethoate	0.67	0.85	0.10	31.70	0.47	0.84	0.07	21.68
					HQs:33.99				HQs: 23.26
10	Dimethoate	0.04	0.81	0.01	0.27	0.03	0.82	0.00	0.19
	Omethoate	0.40	0.85	0.06	18.65	0.27	0.84	0.04	12.75
					HQs:18.92				HQs: 12.94
14	Dimethoate	0.00	0.81	0.00	0.00	0.00	0.82	0.00	0.00
	Omethoate	0.20	0.85	0.03	9.32	0.14	0.84	0.02	6.38
					HQs:9.32				HQs: 6.38

^a^Residues and transfer rates of dimethoate and omethoate on days 5–14 were estimated based on days 1 and 3.

For these made tea samples manufactured on days 1, 3, 5, 7, 10, 14, EDI of dimethoate decreased to 0 from initial value of 3.28–4.85 μg kg^-1^ bw day^-1^, whereas EDI of omethoate varied from 0.02–0.25 μg kg^-1^ bw day^-1^. For black tea, HQs of total residues (both dimethoate and its metabolite) decreased from 211.10 to 6.38 with time interval increasing, following with first-order kinetic (*HQ*_*t*_ = 308.7911 × e ^−0.3210t^,r = 0.9835) with a high correlation coefficient of 0.9835, while for green tea, it ranged from 286.22 to 9.32 (*HQ*_*t*_ = 393.3217 × e ^−0.3104t^, r = 0.9921), which is much higher to black tea manufactured on the same day. HQ value of 10% was considered to be the boundary of safety and risk. HQs < 10% indicated there are no adverse effects on human health following exposure to pesticides [41]. HQs of dimethoate decreased to 1.58–2.28% in made tea on day 7, indicating that risks associated with exposure to dimethoate could be negligible. However, low residue levels of omethoate would contribute a high HQ due to its quite low ADI of 0.3 μg kg^-1^bw day^-1^ (which is less than one fifth that of dimethoate). HQs of omethoate were still higher than 10% on day 7 and decreased to 6.38% and 9.32% on day 14 in black tea and green tea, respectively. Therefore, omethoate was considered to be the most important factor to prolong the safety interval period of dimethoate.

## Conclusion

In this study, the dissipation dynamic and residues for dimethoate and its metabolite during tea plantation, manufacturing, and brewing were investigated. Dimethoate was found to dissipate following the first-order kinetic with a half-life of 1.08 d. The metabolite of omethoate prolonged the half-life of total residues to 1.27 d. Black tea manufacturing has greater effects on pesticides dissipation than green tea manufacturing, wherein drying was the key factor for dissipation in both manufacturing processes. Degradation of dimehtoate increased the concentration of omehtoate during tea cultivation and manufacturing. Most of dimethoate and omethoate in made tea (80.5–84.9%) transferred into the tea infusion easily, attributed to their high water solubility and low octanol-water partition coefficient. From the results of HQs, omethoate demonstrated higher risks than dimethoate to tea drinkers due to its low ADI. These results obtained in this study make sense to instruction for the application of dimethoate formulation on tea crop and global harmonization of MRLs.

## Abbreviations

QuEChERS: quick, easy, cheap, effective,rugged and safe
MRL: maximum residue limit
EC: emulsifiable concentrate
NaCl: sodium chloride
Na_2_SO_4_: anhydrous sodium sulfate
MgSO_4_: anhydrous magnesium sulfate
PSA: primary-secondary amine
GCB: graphitized carbon black
C_18_: octadecylsilane
TRI CAAS: Tea Research Institute, Chinese Academy of Agricultural Sciences
GC: gas chromatography
PF: processing factor
EDI: estimated daily intake
ADI: acceptable daily intake
HQ: hazard quotient
LOQ: limit of quantification
RSD: relative standard deviation
*W*_*s*_: water solubility
*K*_*ow*_: octanol-water partition coefficient
